# Molecular Characterization, Virulence Gene Profiling and Antimicrobial Susceptibility of *Pasteurella multocida* Isolated From Companion Rabbits in Southwestern Iran

**DOI:** 10.1002/vms3.71323

**Published:** 2026-11-12

**Authors:** Darioush Gharibi, Mahdi Pourmahdi Borujeni, Bahman Mosallanejad, Fatemeh Sadat Asadi Khoonsari

**Affiliations:** ^1^ Department of Pathobiology Faculty of Veterinary Medicine Shahid Chamran University of Ahvaz Ahvaz Iran; ^2^ Department of Food Hygiene Faculty of Veterinary Medicine Shahid Chamran University of Ahvaz Ahvaz Iran; ^3^ Department of Clinical Sciences Faculty of Veterinary Medicine Shahid Chamran University of Ahvaz Ahvaz Iran; ^4^ Graduated of Veterinary Medicine Shahid Chamran University of Ahvaz Ahvaz Iran

**Keywords:** antimicrobial resistance, companion rabbits, LPS genotyping, molecular epidemiology, *Pasteurella multocida*, virulence‐associated genes

## Abstract

**Background:**

*Pasteurella multocida* is an important opportunistic pathogen associated with respiratory and systemic diseases in rabbits. Although rabbit pasteurellosis has been extensively investigated in clinically affected animals, limited information is available regarding the molecular characteristics, virulence‐associated genes and antimicrobial susceptibility of *P. multocida* circulating among companion‐rabbit populations.

**Objectives:**

This study investigated the occurrence, molecular characteristics, virulence gene profiles and antimicrobial susceptibility of *P. multocida* isolated from companion rabbits in southwest Iran.

**Methods:**

Nasal and oropharyngeal swabs were collected from 65 companion rabbits, including 62 animals with no apparent clinical abnormalities and three animals with concurrent clinical findings, with no recorded history of antimicrobial treatment before sampling. *P. multocida* was isolated using conventional bacteriological methods and confirmed by species‐specific PCR targeting the kmt1 gene. Confirmed isolates were further characterized by capsular typing, lipopolysaccharide (LPS) genotyping and virulence gene profiling. Antimicrobial susceptibility was evaluated by disk diffusion under CLSI‐referenced testing conditions. Because rabbit‐specific clinical breakpoints for *P. multocida* are unavailable, individual inhibition‐zone diameters were reported as the primary antimicrobial susceptibility testing (AST) outcomes.

**Results:**

*P. multocida* was detected in 6 of 65 sampled rabbits (9.23%). Of the six PCR‐positive rabbits, three were clinically asymptomatic, one had ocular and nasal discharge accompanied by respiratory signs, and two had dental malocclusion with dental abscesses. No statistically significant associations were observed between *P. multocida* carriage and rabbit age, sex or breed (*p* > 0.05). Capsular typing identified five isolates (83.3%) as capsular type A, whereas one isolate (16.7%) was non‐typable. All isolates belonged to LPS genotype L2. The virulence genes *sodA*, *sodC*, *exbD‐tonB* and *nanB* were detected in all isolates (100%). The genes *ompH*, *hgbB*, *pfhA* and *nanH* were detected in four isolates (66.7%), whereas *ptfA*, *oma87*, *hgbA* and *toxA* were identified in three isolates (50%). Disk‐diffusion testing demonstrated variable inhibition‐zone diameters among the tested antimicrobial agents. In the absence of rabbit‐specific clinical breakpoints for *P. multocida*, these findings were reported as in vitro inhibition‐zone measurements rather than rabbit‐specific clinical susceptibility or resistance categories.

**Conclusions:**

The detection of *P. multocida* in both clinically asymptomatic rabbits and rabbits with concurrent clinical abnormalities highlights the heterogeneous clinical context of bacterial carriage and infection in companion rabbits. Molecular surveillance remains important for characterizing circulating strains and improving understanding of rabbit‐associated *P. multocida*. The predominance of capsular type A and LPS genotype L2 provides baseline molecular epidemiological data for companion rabbits in southwest Iran and highlights the importance of continuous molecular surveillance, antimicrobial stewardship and One Health–based disease control strategies.

## Graphical abstract

*Pasteurella multocida* was detected in 6/65 companion rabbits (9.23%), including three asymptomatic rabbits and three with concurrent clinical abnormalities. Five isolates were capsular type A, one was non‐typable, and all six were LPS genotype L2; virulence‐gene profiles varied among isolates. Disk diffusion showed variable inhibition‐zone diameters across 14 antimicrobials; because rabbit‐specific clinical breakpoints are unavailable, results were reported as inhibition‐zone measurements rather than clinical susceptibility/resistance categories.

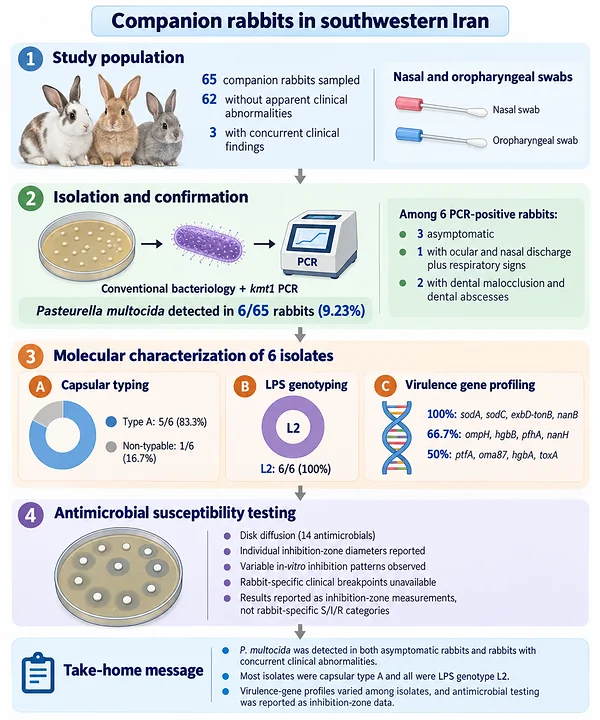

## 1 Introduction

*Pasteurella multocida* is an opportunistic Gram‐negative bacterial pathogen with a broad host range and substantial veterinary importance. It is responsible for a wide spectrum of diseases in domestic and wild animals, causing substantial economic losses, animal welfare concerns and public health implications worldwide (Harper et al. 2006; Wilson and Ho 2013; Peng et al. 2019). In rabbits, *P. multocida* is the principal etiological agent of pasteurellosis, a multifactorial disease that primarily affects the respiratory tract but may also cause conjunctivitis, otitis media, abscesses, reproductive disorders, septicaemia and other systemic infections (Harper et al. 2006; Gleeson and Petritz 2020). The ability of the bacterium to establish persistent colonization in clinically healthy rabbits further increases its epidemiological significance.

Companion rabbits (*Oryctolagus cuniculus*) have become increasingly popular worldwide, resulting in closer contact with humans and other household animals and increasing the importance of infectious disease surveillance in these animals (González‐Martínez et al. 2022; Gleeson and Petritz 2020). Healthy rabbits frequently act as asymptomatic carriers of *P. multocida*, maintaining the organism within the upper respiratory tract for prolonged periods without obvious clinical manifestations (DiGiacomo et al. 1983; Ferreira et al. 2016; Yang et al. 2022). These carrier animals may represent an important source of bacterial transmission within households, breeding facilities and veterinary clinics. Therefore, molecular characterization of *P. multocida* isolates recovered from clinically healthy companion rabbits is essential for understanding bacterial circulation and identifying strains with pathogenic potential.

The pathogenicity of *P. multocida* is mediated by a diverse repertoire of virulence‐associated factors, including the polysaccharide capsule, lipopolysaccharide (LPS), adhesins, neuraminidases, iron acquisition systems, outer membrane proteins and antioxidant enzymes that facilitate colonization, immune evasion and persistence within the host (Ewers et al. 2006; Hatfaludi et al. 2010; Peng et al. 2019). Molecular characterization based on capsular typing, LPS genotyping and virulence‐associated gene profiling has become a cornerstone of epidemiological investigations, providing valuable insights into strain diversity, host adaptation and pathogenic potential (Townsend et al. 2001; Harper, St Michael, et al. 2015; Smith et al. 2021). Recent investigations have demonstrated that combining molecular typing with phenotypic characterization improves epidemiological surveillance and facilitates comparison of circulating strains among geographical regions (D'Amico et al. 2024; Ziagham et al. 2024).

Antimicrobial therapy remains an important component of rabbit pasteurellosis management; however, the emergence of antimicrobial‐resistant *P. multocida* strains has become an increasing concern in veterinary medicine. Antimicrobial susceptibility profiles vary considerably according to geographical region, host species and antimicrobial usage practices, emphasizing the importance of continuous regional surveillance to support evidence‐based therapeutic decisions and antimicrobial stewardship (Tang et al., 2009; Wilson and Ho, 2013; Michael et al. 2018; Bourély et al. 2019; Nielsen et al., 2021; Haulisah et al. 2022). Because companion rabbits frequently live in close contact with humans and other household animals, monitoring antimicrobial resistance in *P. multocida* also has relevance within the One Health framework (Supporting Information).

Although molecular epidemiological studies have characterized *P. multocida* isolates from livestock, poultry, diseased rabbits and other companion animals, comprehensive studies simultaneously evaluating capsular genotype, LPS genotype, virulence‐associated genes and antimicrobial susceptibility among companion‐rabbit isolates remain limited. In Iran, previous molecular investigations have primarily focused on isolates recovered from food‐producing animals, whereas comparable information regarding companion rabbits remains scarce (Jabbari et al. 2014; Gharib Mombeni et al. 2021; Tabatabaei and Abdolahi 2023). Therefore, additional molecular epidemiological data are required to improve understanding of circulating *P. multocida* strains in companion rabbits.

Accordingly, the present study aimed to determine the occurrence of *P. multocida* among companion rabbits in southwest Iran and to characterize the recovered isolates with respect to capsular genotype, LPS genotype, virulence‐associated gene profile and antimicrobial susceptibility. The findings provide baseline molecular epidemiological data for companion rabbits in Iran and contribute to improved surveillance, evidence‐based antimicrobial use and One Health–oriented control strategies for rabbit‐associated *P. multocida*.

## 2 Materials and Methods

### 2.1 Study Design and Sample Collection

This cross‐sectional study was conducted between February 2023 and January 2024 in Ahvaz, Khuzestan Province, southwest Iran. A total of 65 companion rabbits (*O. cuniculus*) were sampled from five accessible sources during the study period: 26 rabbits (40.0%) presented to the Veterinary Teaching Hospital/Faculty of Veterinary Medicine, Shahid Chamran University of Ahvaz; 21 rabbits (32.3%) were maintained in local pet shops in Ahvaz; 8 rabbits (12.3%) were maintained in the grounds of the female student dormitory of Shahid Chamran University of Ahvaz; 6 rabbits (9.2%) were maintained in the Parasitology Section of the Faculty of Veterinary Medicine; and 4 rabbits (6.2%) were maintained in the Faculty of Veterinary Medicine animal house. Thus, recruitment was source‐based and non‐population‐based rather than a probability sample of the regional companion‐rabbit population.

A formal a priori sample‐size calculation was not performed; the sample size reflected the rabbits available for inclusion from these sources during the predefined study period. Accordingly, the study was intended to describe the occurrence and characteristics of *P. multocida* within the sampled population rather than to provide a population‐representative estimate for all companion rabbits in southwest Iran.

Of the 65 rabbits, 62 had no apparent clinical abnormalities at the time of sampling, whereas three presented concurrent clinical findings: one rabbit had ocular and nasal discharge accompanied by respiratory signs, and two rabbits had dental malocclusion with dental abscesses. The presence of these abnormalities did not constitute an exclusion criterion. Demographic and clinical information, including age, sex, breed, source/housing, disease history and previous antimicrobial use, was recorded before sampling.

Following informed owner consent, paired nasal and oropharyngeal swab samples were aseptically collected from each rabbit using sterile cotton swabs. Both swabs obtained from each animal were inoculated onto the same primary culture plate to maximize the recovery of *P. multocida*. Immediately after collection, the samples were transported to the Department of Microbiology Laboratory under refrigerated conditions and processed within 2 h.

### 2.2 Bacterial Isolation and Phenotypic Identification

Samples were inoculated onto 5% sheep blood agar (Merck, Germany) using the quadrant streak method and incubated aerobically at 37°C for 24–48 h. Colonies exhibiting morphological characteristics consistent with *P. multocida* (small, circular, smooth, greyish and non‐haemolytic colonies) were selected for further examination.

Presumptive isolates were characterized by Gram staining and standard biochemical tests, including catalase and oxidase activity, growth on MacConkey agar, indole production, urease activity and triple sugar iron (TSI) agar reactions, according to standard microbiological procedures (Bryan et al. 2013). Isolates showing biochemical characteristics consistent with *P. multocida* were preserved at −70°C in glycerol‐containing storage medium until molecular characterization and antimicrobial susceptibility testing (AST).

### 2.3 Molecular Identification and Characterization

#### 2.3.1 DNA Extraction

Crude DNA extract was extracted from presumptive *P. multocida* isolates using the boiling lysis method (Dashti et al. 2009). Briefly, a single bacterial colony was suspended in 200 µL of sterile distilled water and incubated at 100°C for 10 min. The suspension was centrifuged at approximately 1680 × *g* (5000 rpm) for 5 min, and the supernatant containing crude genomic DNA was collected and stored at −20°C until PCR analysis.

#### 2.3.2 Species Confirmation by PCR

Presumptive isolates were confirmed as *P. multocida* by PCR amplification of the species‐specific kmt1 gene using the primers listed in Table 1. PCR was performed in a final volume of 25 µL containing 12.5 µL of 2× PCR Master Mix (Ampliqon, Denmark), 2.2 µL of each primer (10 µM), 4 µL of template DNA and 4.1 µL of nuclease‐free water. Amplification consisted of initial denaturation at 95°C for 5 min, followed by 35 cycles of denaturation at 95°C for 60 s, annealing at 55°C for 45 s and extension at 72°C for 40 s, followed by a final extension at 72°C for 10 min (Townsend et al. 1998). DNA from *P. multocida* ATCC 43137 was used as the positive control. A negative control was included in each PCR run to monitor contamination. The PCR reaction composition and thermal cycling conditions are summarized in Tables 2 and 3.

**TABLE 1:** Primers used for molecular identification, capsular and LPS genotyping and detection of virulence‐associated genes.

Target	Gene	Forward primer (5′–3′)	Reverse primer (5′–3′)	Expected amplicon size (bp)	Reference
Species identification	*kmt1*	ATCCGCTATTTACCCAGTGG	GCTGTAAACGAACTCGCCAC	460	Townsend et al. (1998)
Capsular genotype A	*hyaD–hyaC*	GATGCCAAAATCGCAGTCAG	TGTTGCCATCATTGTCAGTG	1048	Townsend et al. (2001)
Capsular genotype B	*BcbD*	CATTTATCCAAGCTCCACC	GCCCGAGAGTTTCAATCC	758	Townsend et al. (2001)
Capsular genotype D	*dcbF*	TTACAAAAGAAAGACTAGGAGCCC	CATCTACCCACTCAACCATATCAG	657	Townsend et al. (2001)
Capsular genotype E	*ecbJ*	TCCGCAGAAAATTATTGACTC	GCTTGCTGCTTGATTTTGTC	511	Townsend et al. (2001)
Capsular genotype F	*fcbD*	AATCGGAGAACGCAGAAATCAG	TTCCGCCGTCAATTACTCTG	851	Townsend et al. (2001)
LPS genotype L1	*pcgD‐B*	ACATTCCAGATAATACACCCG	ATTGGAGCACCTAGTAACCC	1307	Harper, John, et al. (2015), Harper, St Michael, et al. (2015)
LPS genotype L2	*nctA*	CTTAAAGTAACACTCGCTATTGC	TTTGATTTCCCTTGGGATAGC	810	Harper, John, et al. (2015), Harper, St Michael, et al. (2015)
LPS genotype L3	*gatF*	TGCAGGCGAGAGTTGATAAACCATC	CAAAGATTGGTTCCAAATCTGAATGGA	474	Harper, John, et al. (2015), Harper, St Michael, et al. (2015)
LPS genotype L4	*latB*	TTTCCATAGATTAGCAATGCCG	CTTTATTTGGTCTTTATATATACC	550	Harper, John, et al. (2015), Harper, St Michael, et al. (2015)
LPS genotype L5	*rmlA‐C*	AGATTGCATGGCGAAATGGC	CAATCCTCGTAAGACCCCC	1175	Harper, John, et al. (2015), Harper, St Michael, et al. (2015)
LPS genotype L6	*nctB*	TCTTTATAATTATACTCTCCCAAGG	AATGAAGGTTTAAAAGAGATAGCTGGAG	668	Harper, John, et al. (2015), Harper, St Michael, et al. (2015)
LPS genotype L7	*ppgB*	CCTATATTTATATCTCCTCCCC	CTAATATATAAACCATCCAACGC	931	Harper, John, et al. (2015), Harper, St Michael, et al. (2015)
LPS genotype L8	*natG*	GAGAGTTACAAAAATGATCGGC	TCCTGGTTCATATATAGGTAGG	255	Harper, John, et al. (2015), Harper, St Michael, et al. (2015)
Adhesion	*ptfA*	TGTGGAATTCAGCATTTTAGTGTGTC	TCATGAATTCTTATGCGCAAAATCCTGCTGG	488	Doughty et al. (2000)
	*pfhA*	AGCTGATCAAGTGGTGAAC	TGGTACATTGGTGAATGCTG	275	Ewers et al. (2006)
Outer membrane protein	*ompH*	CGCGTATGAAGGTTTAGGT	TTTAGATTGTGCGTAGTCAAC	438	Ewers et al. (2006)
	*oma87*	ATGAAAAAACTTTTAATTGCGAGC	TGACTTGCGCAGTTGCATAAC	948	Ewers et al. (2006)
Iron acquisition system	*hgbA*	TGGCGGATAGTCATCAAG	CCAAAGAACCACTACCCA	419	Ewers et al. (2006)
	*hgbB*	ACCGCGTTGGAATTATGATTG	CATTGAGTACGGCTTGACAT	788	Atashpaz et al. (2009)
	*exbD–tonB*	GGTGGTGATATTGATGCGGC	GCATCATGCGTGCACGGTT	1144	Ewers et al. (2006)
Oxidative stress response	*sodA*	TACCAGAATTAGGCTACGC	GAAACGGGTTGCTGCCGCT	361	Ewers et al. (2006)
	*sodC*	AGTTAGTAGCGGGGTTGGCA	TGGTGCTGGGTGATCATCATG	235	Lainson et al. (1996)
Sialidase	*nanB*	GTCCTATAAAGTGACGCCGA	ACAGCAAAGGAAGACTGTCC	554	Ewers et al. (2006)
	*nanH*	GAATATTTGGGCGGCAACA	TTCTCGCCCTGTCATCACT	360	Ewers et al. (2006)
Dermonecrotic toxin	*toxA*	TCTTAGATGAGCGACAAGG	GAATGCCACACCTCTATAG	865	Lichtensteiger et al. (1996)

Abbreviation: LPS, lipopolysaccharide.

**TABLE 2:** Composition of PCR reaction mixtures used for molecular identification, capsular and LPS genotyping and virulence gene detection.

PCR assay	PCR Master Mix (µL)	Forward primers (10 µM) (µL)	Reverse primers (10 µM) (µL)	Template DNA (µL)	Nuclease‐free water (µL)	Final volume (µL)
Species‐specific PCR (*kmt1*)	12.5	2.2	2.2	4	4.1	25
Capsular multiplex PCR	25	2.0 each × 5 (10 total)	2.0 each × 5 (10 total)	4	1	50
LPS multiplex PCR	25	0.25 each × 8 (2 total)	0.25 each × 8 (2 total)	5	16	50
Virulence gene multiplex PCR	12.5	0.5 each × 4 (2 total)	0.5 each × 4 (2 total)	4	4.5	25

Abbreviation: LPS, lipopolysaccharide.

**TABLE 3:** Thermal cycling conditions used for molecular identification, capsular and LPS genotyping and virulence gene detection.

PCR assay	Initial denaturation	PCR cycling	Final extension
Species‐specific PCR (*kmt1*)	95°C for 5 min	95°C for 60 s; 55°C for 45 s; 72°C for 40 s	72°C for 10 min
Capsular multiplex PCR	95°C for 5 min	95°C for 60 s; 55°C for 60 s; 72°C for 60 s	72°C for 10 min
LPS multiplex PCR	95°C for 5 min	95°C for 30 s; 54°C for 30 s; 72°C for 45 s	72°C for 10 min
Virulence gene multiplex PCR	95°C for 5 min	94°C for 30 s; 55°C for 30 s; 72°C for 60 s	72°C for 10 min

Abbreviation: LPS, lipopolysaccharide.

#### 2.3.3 Capsular Genotyping

Confirmed *P. multocida* isolates were subjected to multiplex PCR for capsular genotyping (capsular types A, B, D, E and F) described by Townsend et al. (2001). Each reaction was performed in a final volume of 50 µL, containing 25 µL of 2× PCR Master Mix, 2 µL of each forward and reverse capsular primer (five primer pairs; 20 µL total primer volume), 4 µL of template DNA and 1 µL of nuclease‐free water. Amplification consisted of initial denaturation at 95°C for 5 min, followed by 35 cycles of 95°C for 60 s, 55°C for 60 s and 72°C for 60 s, with a final extension at 72°C for 10 min. DNA from *P. multocida* ATCC 43137 and strain HN06 was used as positive‐control material for capsular genotyping. A negative control was included in each PCR run. Isolates that could not be assigned to a capsular genotype by multiplex PCR were additionally examined using individual capsular PCR assays.

#### 2.3.4 LPS Genotyping

LPS genotypes L1–L8 were determined the using multiplex PCR assay developed by Harper, John, et al. (2015), Harper, St Michael, et al. (2015). Each reaction was performed in a final volume of 50 µL containing 25 µL of PCR Master Mix, 0.25 µL of each primer (eight primer pairs; 4 µL total primer volume), 5 µL of template DNA and 16 µL of nuclease‐free water. Amplification consisted of initial denaturation at 95°C for 5 min, followed by 30 cycles of denaturation at 94°C for 30 s, annealing at 54°C for 30 s and extension at 72°C for 45 s, followed by a final extension at 72°C for 10 min. DNA from *P. multocida* ATCC 43137 and ATCC 2100 was used as positive‐control material for the LPS genotyping assay. A negative control was included in each PCR run. PCR reaction mixtures and amplification conditions are summarized in Tables 2 and 3.

#### 2.3.5 Detection of Virulence‐Associated Genes

The presence of 12 virulence‐associated genes representing adhesins (*ptfA* and *pfhA*), iron acquisition systems (*hgbA*, *hgbB* and *exbD‐tonB*), outer membrane proteins (*ompH* and *oma87*), neuraminidases (*nanB* and *nanH*), superoxide dismutases (*sodA* and *sodC*) and dermonecrotic toxin (*toxA*) was investigated by multiplex PCR.

The target genes were amplified in three multiplex PCR assays, each including four virulence genes as follows: Multiplex I: *sodA, hgbA, ptfA* and *pfhA;* Multiplex II: *ompH, exbD‐tonB, nanH* and *toxA* and Multiplex III: *oma87, sodC, hgbB* and *nanB*.

Each PCR reaction was carried out in a final volume of 25 µL containing 12.5 µL of 2× PCR Master Mix (Ampliqon, Denmark), 0.5 µL of each primer (10 µM), 4 µL of DNA template, and nuclease‐free water to the final reaction volume. The primer sequences and thermal cycling conditions are provided in Tables 1, 2, 3. The multiplex PCR protocol was performed according to the methods described by Gharib Mombeni et al. (2021) and Ziagham et al. (2024). Positive‐control DNA from *P. multocida* ATCC 12945, ATCC 43137 and strain HN06 was included in the virulence‐gene multiplex PCR assays. Negative controls were included in each PCR run to monitor contamination.

#### 2.3.6 Agarose Gel Electrophoresis

PCR amplicons were analysed by electrophoresis on 1.5% agarose gels (Sinaclon Co. Tehran, Iran) prepared in 1× TAE buffer and stained with Safe Stain (Sinaclon Co. Tehran, Iran). Aliquots of 8 µL of each PCR product were loaded onto the gel together with either a 50 bp or a 100 bp DNA ladder, depending on the expected amplicon size. Electrophoresis was performed at 100 V until adequate separation of DNA fragments was achieved. PCR products were visualized and photographed under ultraviolet (UV) illumination using a gel documentation system.

### 2.4 Antimicrobial Susceptibility Testing

The AST of all confirmed *P. multocida* isolates was performed using the Kirby–Bauer disk‐diffusion method on Mueller–Hinton agar supplemented with 5% defibrinated sheep blood. Bacterial suspensions prepared from fresh cultures were adjusted to a 0.5 McFarland standard and uniformly inoculated onto the agar surface. Antimicrobial disks were applied within 15 min, and plates were incubated aerobically at 37°C for 24 h. Following incubation, inhibition‐zone diameters were measured in millimetres. The antimicrobial agents tested were penicillin G (10 U), ceftriaxone (30 µg), tetracycline (30 µg), erythromycin (15 µg), florfenicol (30 µg), lincomycin–spectinomycin (15/100 µg), ampicillin (10 µg), streptomycin (10 µg), ceftiofur (30 µg), tilmicosin (15 µg), trimethoprim–sulphamethoxazole (1.25/23.75 µg), enrofloxacin (5 µg), cefazolin (30 µg) and ceftizoxime (30 µg).

Disk‐diffusion testing conditions were selected with reference to CLSI VET01/VET01S recommendations (CLSI 2024a, 2024b). Because infection‐ and host‐specific clinical breakpoints for *P. multocida* isolated from rabbits are unavailable, the individual inhibition‐zone diameters were considered the primary AST data and were not converted into rabbit‐specific susceptible, intermediate or resistant categories. Where CLSI VET01S‐Ed7 provides disk‐diffusion clinical breakpoints for *P. multocida* associated with respiratory disease in cattle or swine, these criteria are presented in Table 6 for reference only, together with the corresponding host and clinical indication, and were not considered validated predictors of therapeutic response in rabbits. For agents lacking an applicable disk‐diffusion clinical breakpoint, the results were designated as not interpreted (NI) (CLSI 2024a, 2024b; Nielsen et al. 2021; Krüger‐Haker et al. 2026).

### 2.5 Statistical Analysis

Statistical analyses were performed using IBM SPSS Statistics for Windows, version 22.0 (IBM Corp., Armonk, NY, USA). Fisher's exact test was used to evaluate the association between *P. multocida* detection and host‐related variables, including age, sex and breed. All statistical tests were two‐tailed, and a *p* value < 0.05 was considered statistically significant. The 95% confidence interval (CI) for the observed frequency of *P. multocida* detection was calculated using the exact binomial (Clopper–Pearson) method. Because only six *P. multocida*‐positive isolates were available for molecular characterization, virulence‐gene distributions were summarized descriptively, and no inferential statistical comparisons between virulence‐gene profiles and host variables were performed.

## 3 Results

### 3.1 Characteristics of the Study Population and Bacteriological Findings

A total of 65 companion rabbits were included in the study. Sixty‐two rabbits (95.4%) had no apparent clinical abnormalities at the time of sampling, whereas three rabbits (4.6%) presented concurrent clinical findings. One rabbit had ocular and nasal discharge accompanied by respiratory signs, and two rabbits had dental malocclusion with dental abscesses. The population comprised 41 females and 24 males, ranging in age from 2 weeks to 7.5 years. According to breed, 45 rabbits were mixed breed, 14 were Lop, and 6 were New Zealand White.

Primary culture of nasal and oropharyngeal swab samples on 5% sheep blood agar yielded small, smooth, convex, greyish, non‐haemolytic colonies suggestive of *P. multocida*. A total of 11 isolates exhibited colony morphology compatible with *P. multocida* and were selected for further phenotypic characterization.

All 11 presumptive isolates were Gram‐negative coccobacilli and showed biochemical characteristics consistent with *P. multocida*, including positive oxidase and catalase reactions, no growth on MacConkey agar, an acid/acid reaction on TSI agar without hydrogen sulphide production, negative urease activity, negative lactose and maltose fermentation and positive fermentation of glucose, sucrose and mannitol.

Subsequent molecular confirmation by PCR targeting the species‐specific *kmt1* gene identified six isolates (9.23%; 6/65) as *P. multocida*, whereas the remaining five presumptive isolates were excluded from further analyses because they were PCR‐negative.

### 3.2 Molecular Confirmation of *Pasteurella multocida*

PCR amplification targeting the species‐specific *kmt1* gene confirmed 6 of the 11 presumptive isolates as *P. multocida*. Figure 1 shows a representative gel containing two of the six *kmt1*‐positive isolates together with the positive and negative controls; therefore, the number of sample lanes shown in the figure does not represent the total number of isolates analysed. The observed frequency of *P. multocida* detection in the sampled population was 9.23% (6/65; exact 95% CI, 3.46%–19.02%). The remaining five presumptive isolates were negative for *kmt1* and were excluded from subsequent molecular characterization and AST.

**FIGURE 1:**
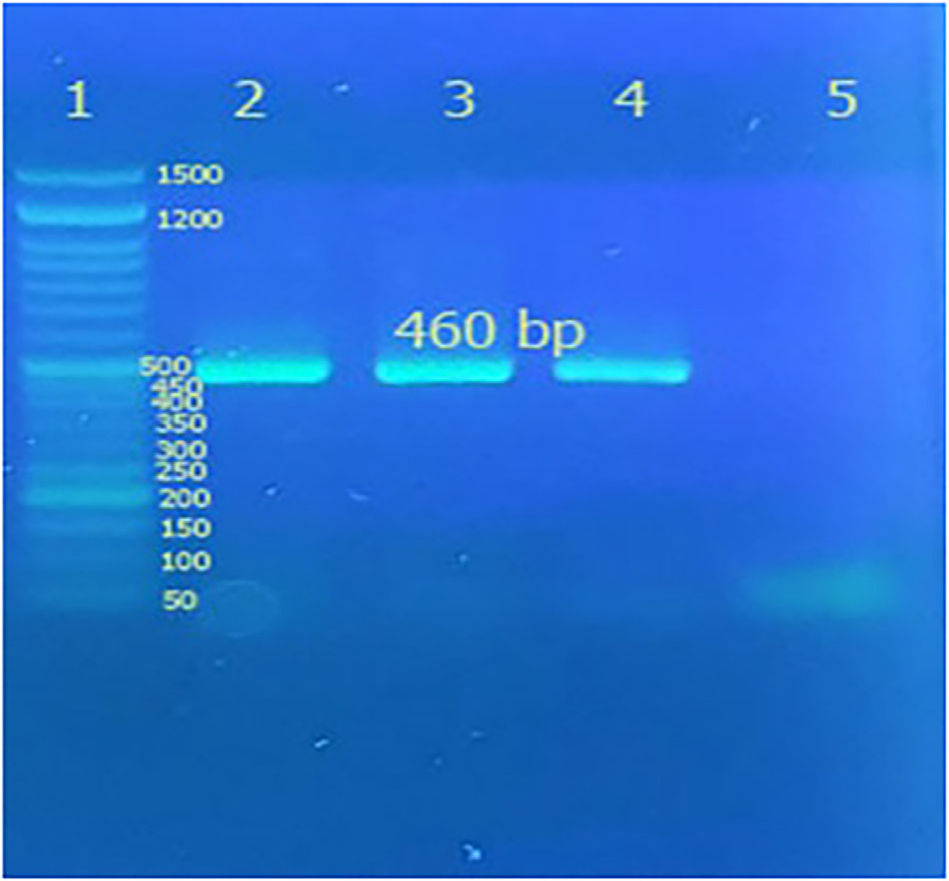
Representative agarose gel electrophoresis of PCR amplification of the species‐specific *kmt1* gene (460 bp) used for molecular confirmation of *Pasteurella multocida*. All 11 presumptive isolates were tested, of which six were *kmt1*‐positive; only two representative positive isolates are shown in this gel. Lane 1: 50‐bp DNA ladder; Lane 2: positive control (*P. multocida* ATCC 43137); Lanes 3 and 4: representative *kmt1*‐positive isolates; Lane 5: negative control.

Among the six PCR‐confirmed *P. multocida*–positive rabbits, three (3/6, 50.0%) had no apparent clinical signs at the time of sampling and were therefore considered asymptomatic carriers. The remaining three positive rabbits had concurrent clinical abnormalities: one rabbit had ocular and nasal discharge accompanied by respiratory signs, and two rabbits had dental malocclusion with dental abscesses. All three rabbits with recorded clinical abnormalities were *P. multocida*–positive.

### 3.3 Association Between *Pasteurella Multocida* Isolation and Host Characteristics

The association between *P. multocida* detection and host‐related variables, including age, sex and breed, was evaluated (Table 4). Of the six PCR‐confirmed isolates, five (83.3%) were recovered from rabbits younger than 1 year of age, whereas one isolate (16.7%) was obtained from a rabbit older than 3 years. The frequency of *P. multocida* detection was 11.63% (5/43) in rabbits <1 year of age, 0% (0/11) in rabbits aged 1–3 years and 9.09% (1/11) in rabbits >3 years of age.

**TABLE 4:** Association between *Pasteurella multocida* isolation and host characteristics of companion rabbits.

Host variable	Category	Positive, *n* (%)	Negative, *n* (%)	Total (*n*)	*p* value
**Age**	<1 year	5 (11.63)	38 (88.37)	43	0.43^*^
	1–3 years	0 (0.00)	11 (100.00)	11	
	>3 years	1 (9.09)	10 (90.91)	11	
**Sex**	Male	2 (8.33)	22 (91.67)	24	1.00^*^
	Female	4 (9.76)	37 (90.24)	41	
**Breed**	Mixed	4 (8.89)	41 (91.11)	45	0.59^*^
	Lop	2 (14.29)	12 (85.71)	14	
	New Zealand White	0 (0.00)	6 (100.00)	6	
**Overall**		**6 (9.23)**	**59 (90.77)**	**65**	—

* *p* values were calculated using Fisher's exact test.

The detection frequency of *P. multocida* was 9.76% (4/41) among female rabbits and 8.33% (2/24) among male rabbits.

Breed‐specific detection frequencies were 8.89% (4/45) in mixed‐breed rabbits, 14.29% (2/14) in Lop rabbits and 0% (0/6) in New Zealand White rabbits.

Fisher's exact test showed no statistically significant association between *P. multocida* isolation and age, sex or breed (*p* > 0.05 for all comparisons).

### 3.4 Capsular Genotyping

Multiplex PCR‐based capsular genotyping identified capsular type A in five (83.3%) of the six *P. multocida* isolates. Isolate 4 (16.7%) produced no type‐specific amplicon corresponding to capsular genotypes A, B, D, E or F and was therefore classified as non‐typable (NT) (Figure 2; Table 5).

**FIGURE 2:**
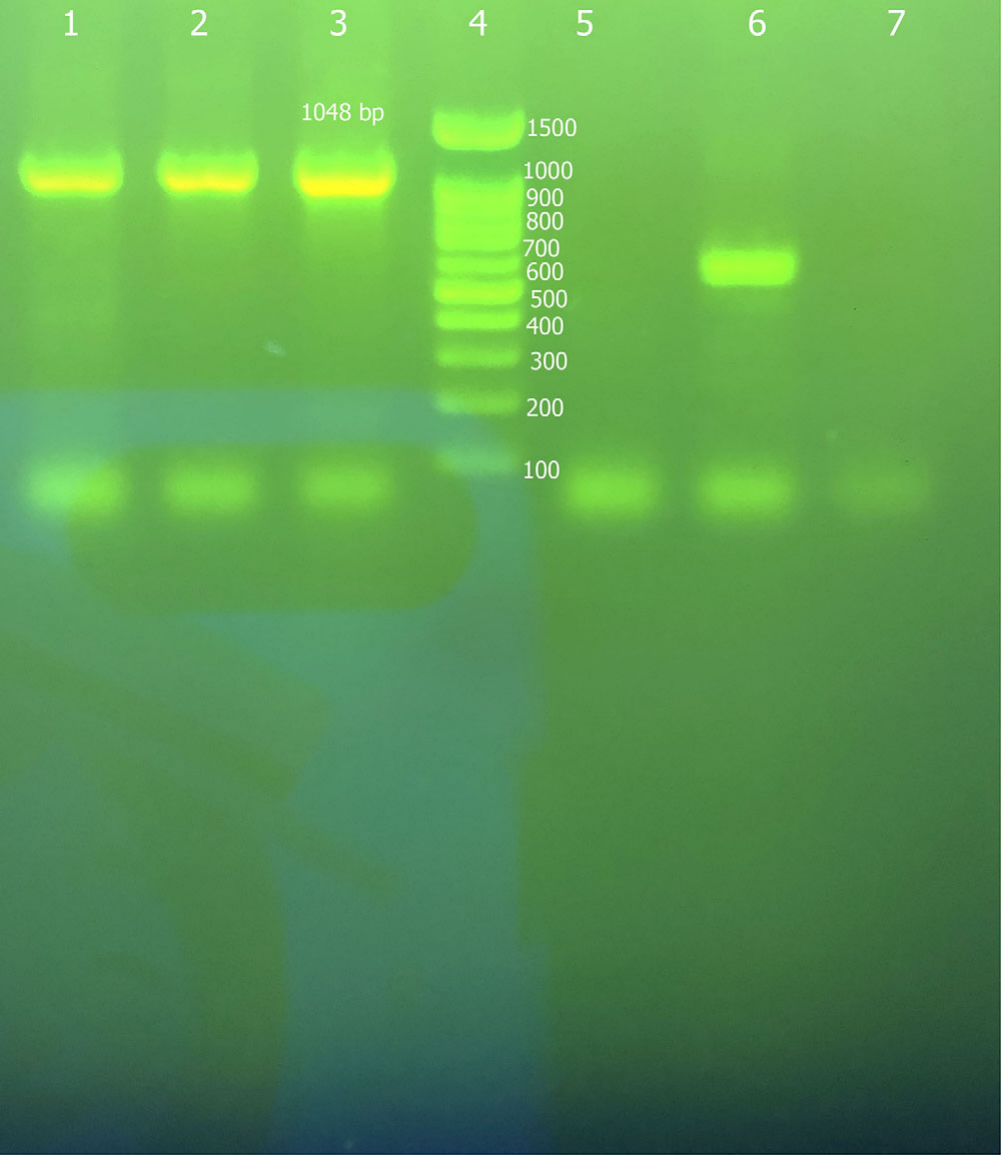
Representative agarose gel electrophoresis of multiplex PCR for capsular genotyping of *Pasteurella multocida*. All six confirmed isolates were subjected to capsular genotyping; selected representative isolates are shown. The 1048‐bp amplicon corresponds to capsular genotype A. Lane 1: positive control for capsular genotype A; Lanes 2 and 3: representative capsular type‐A isolates; Lane 4: 100‐bp DNA ladder; Lane 5: representative non‐typable isolate, with no type‐specific capsular amplicon detected; Lane 6: positive control for capsular genotype D; Lane 7: negative control.

**TABLE 5:** Capsular genotype, LPS genotype and virulence‐associated gene profiles of the six *Pasteurella multocida* isolates.

Isolate ID	Capsular genotype	LPS genotype	ptfA	pfhA	ompH	oma87	hgbA	hgbB	exbD‐tonB	sodA	sodC	nanB	nanH	toxA
1	A	L2	+	+	+	+	+	−	+	+	+	+	+	+
2	A	L2	+	−	+	−	−	+	+	+	+	+	+	−
3	A	L2	−	−	−	+	+	−	+	+	+	+	−	−
4	NT	L2	−	+	+	−	+	+	+	+	+	+	+	+
5	A	L2	+	+	−	−	−	+	+	+	+	+	−	−
6	A	L2	−	+	+	+	−	+	+	+	+	+	+	+
Positive isolates, *n* (%)	A: 5 (83.3%); NT: 1 (16.7%)	L2: 6 (100%)	3 (50%)	4 (66.7%)	4 (66.7%)	3 (50%)	3 (50%)	4 (66.7%)	6 (100%)	6 (100%)	6 (100%)	6 (100%)	4 (66.7%)	3 (50%)

*Note*: +, gene detected; −, gene not detected; NT, non‐typable. Results are shown for all six PCR‐confirmed *P. multocida* isolates; Figures 2, 3, 4, 5, 6 contain representative PCR products from selected isolates only.

Abbreviation: LPS, lipopolysaccharide.

### 3.5 LPS Genotyping

Multiplex PCR‐based LPS genotyping showed that all six *P. multocida* isolates (100%) belonged to LPS genotype L2 (Figure 3; Table 5).

**FIGURE 3:**
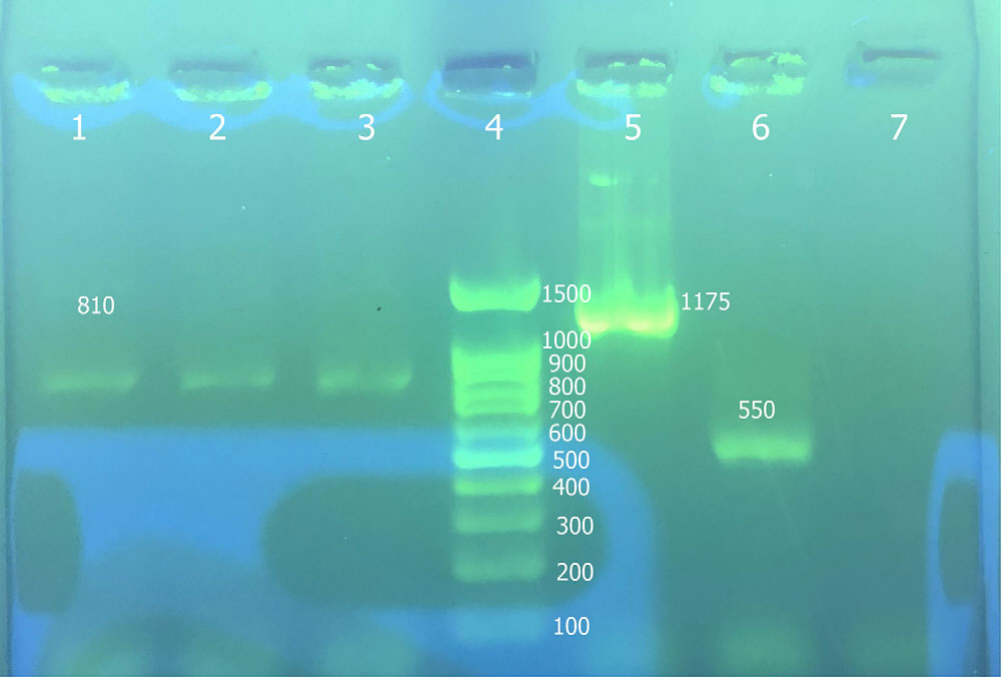
Representative agarose gel electrophoresis of multiplex PCR for lipopolysaccharide (LPS) genotyping of *Pasteurella multocida*. All six confirmed isolates were tested and were identified as LPS genotype L2; selected representative isolates are shown. The 810‐bp amplicon corresponds to LPS genotype L2. Lane 1: positive control for LPS genotype L2; Lanes 2 and 3: representative L2‐positive isolates; Lane 4: 100‐bp DNA ladder; Lane 5: positive control for LPS genotype L5; Lane 6: positive control for LPS genotype L4; Lane 7: negative control.

### 3.6 Distribution of Virulence‐Associated Genes

All six *P. multocida* isolates harboured the sodA, exbD‐tonB, nanB and sodC genes (100%). The ompH, hgbB, pfhA and nanH genes were detected in four isolates (66.7%), whereas ptfA, oma87, hgbA and toxA were each identified in three isolates (50%). The complete capsular genotype, LPS genotype and isolate‐level virulence‐gene profiles of all six isolates are presented in Table 5. Figures 2, 3, 4, 5, 6 contain representative PCR products from selected isolates and therefore do not display all six isolates simultaneously; Figures 4, 5, 6 specifically show representative multiplex‐PCR gel images for the virulence‐associated genes. The complete positive/negative molecular profile of each isolate is provided in Table 5.

**FIGURE 4:**
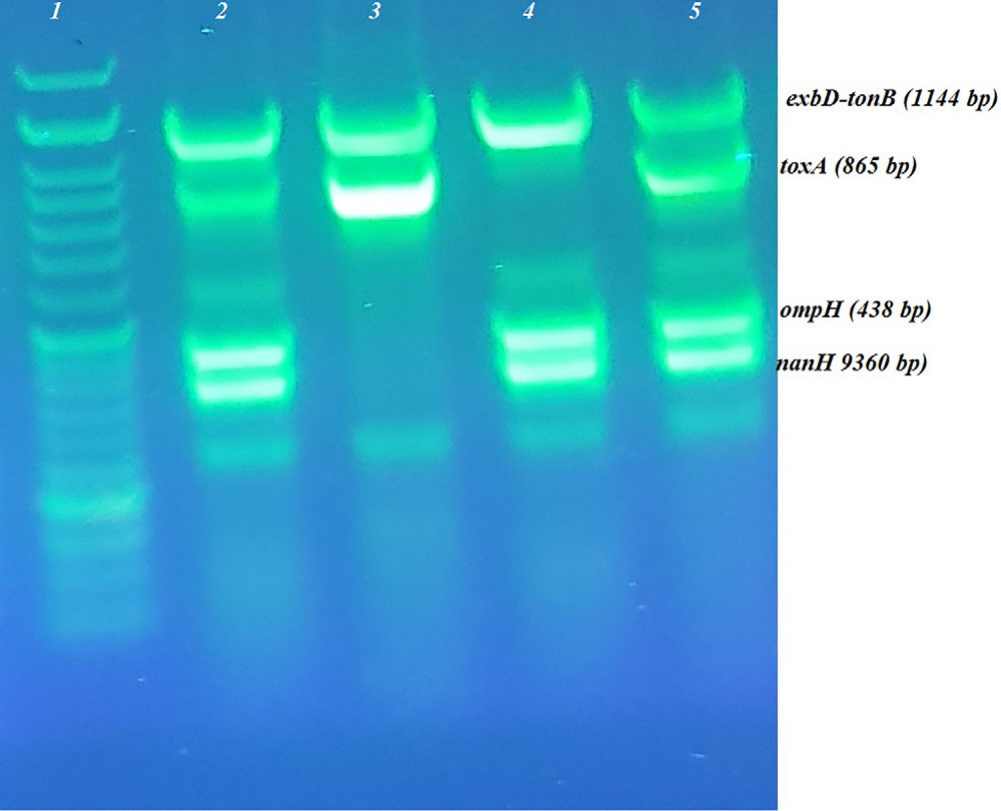
Representative agarose gel electrophoresis of multiplex PCR amplification of the virulence‐associated genes *exbD‐tonB*, *toxA*, *ompH* and *nanH*. Expected amplicon sizes were 1144 bp for *exbD‐tonB*, 865 bp for *toxA*, 438 bp for *ompH* and 360 bp for *nanH*. Lane 1: 50‐bp DNA ladder; Lane 2: positive control; Lanes 3–5: representative PCR products from selected *Pasteurella multocida* isolates showing different amplification profiles for the four target genes. A band at the expected amplicon size was considered positive for the corresponding gene, whereas absence of the expected band was considered negative. The gel image is representative and does not include all six isolates analysed. Complete isolate‐level positive/negative results for all six isolates are presented in Table 5.

**FIGURE 5:**
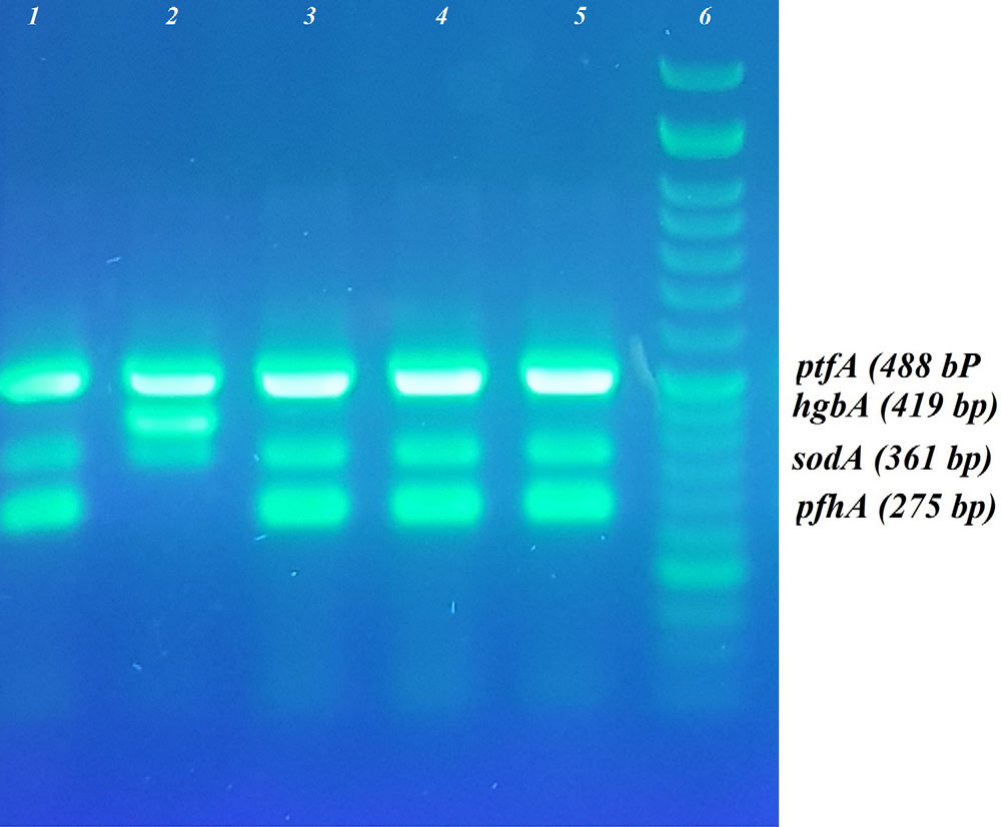
Representative agarose gel electrophoresis of multiplex PCR amplification of the virulence‐associated genes *ptfA*, *sodA*, *pfhA* and *hgbA*. Expected amplicon sizes were 488 bp for *ptfA*, 361 bp for *sodA*, 275 bp for *pfhA* and 419 bp for *hgbA*. Lanes 1–4: representative PCR products from selected *Pasteurella multocida* isolates showing different amplification profiles for the four target genes; Lane 5: positive control; Lane 6: 50‐bp DNA ladder. A band at the expected amplicon size was considered positive for the corresponding gene, whereas absence of the expected band was considered negative. The gel image is representative and does not include all six isolates analysed. Complete isolate‐level positive/negative results for all six isolates are presented in Table 5.

**FIGURE 6:**
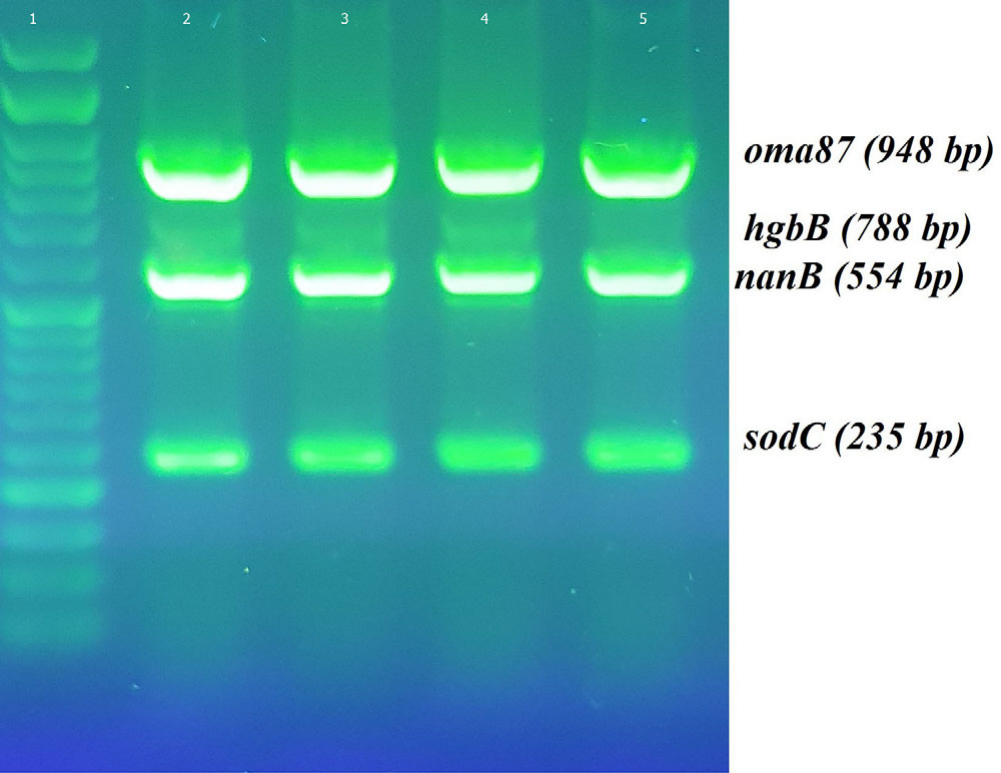
Representative agarose gel electrophoresis of multiplex PCR amplification of the virulence‐associated genes *oma87*, *hgbB*, *nanB* and *sodC*. Lane 1: 50‐bp DNA ladder; Lane 2: positive control; Lanes 3–5: representative PCR products from selected *Pasteurella multocida* isolates showing different amplification profiles for the four target genes. A band at the expected amplicon size was considered positive for the corresponding gene, whereas absence of the expected band was considered negative. The gel image is representative and does not include all six isolates analysed. Complete isolate‐level positive/negative results for all six isolates are presented in Table 5.

### 3.7 Antimicrobial Susceptibility Testing

Disk‐diffusion testing generated a wide range of inhibition‐zone diameters among the antimicrobial agents tested against the six *P. multocida* isolates (Table 6). Because rabbit‐specific clinical breakpoints are not available for *P. multocida*, the individual inhibition‐zone diameters are presented as the primary AST results, and rabbit‐specific susceptible, intermediate or resistant categories were not assigned.

**TABLE 6:** Individual inhibition‐zone diameters of the six *Pasteurella multocida* isolates and the availability of clinical interpretive criteria.

Antimicrobial agent (disk content)	Isolate 1	Isolate 2	Isolate 3	Isolate 4	Isolate 5	Isolate 6	CLSI VET01S interpretive context
Penicillin G (10 U)	18	32	15	20	25	19	**NI**; no rabbit‐specific DD CBP. For bovine/swine respiratory *P. multocida*, CLSI criteria are MIC‐based only
Ceftriaxone (30 µg)	33	44	30	29	30	36	**NI**; no applicable rabbit‐specific *P. multocida* DD CBP
Tetracycline (30 µg)	36	35	25	28	35	30	**NI**; no rabbit‐specific DD CBP. Bovine/swine respiratory *P. multocida* criteria are MIC‐based only
Erythromycin (15 µg)	22	28	12	15	15	20	**NI**; no applicable rabbit‐specific *P. multocida* DD CBP
Florfenicol (30 µg)	32	38	30	32	35	38	Rabbit CBP is unavailable. Reference only: cattle respiratory *P. multocida*: *S* ≥ 19, *I* 15–18, *R* ≤ 14 mm; swine: *S* ≥ 22, *I* 19–21, *R* ≤ 18 mm
Lincomycin–spectinomycin (15/100 µg)	36	50	32	35	44	32	**NI**; no validated rabbit‐specific DD CBP for this combination
Ampicillin (10 µg)	22	36	25	26	26	23	**NI**; no rabbit‐specific DD CBP. Bovine/swine respiratory *P. multocida* criteria are MIC‐based only
Streptomycin (10 µg)	10	18	23	15	14	10	**NI**; no applicable rabbit‐specific *P. multocida* DD CBP
Ceftiofur (30 µg)	29	27	27	10	10	13	Rabbit CBP is unavailable. Reference only: cattle/swine respiratory *P. multocida*: *S* ≥ 21, *I* 18–20, *R* ≤ 17 mm
Tilmicosin (15 µg)	16	9	0*	0*	0*	0*	Rabbit CBP is unavailable. Reference only: swine respiratory *P. multocida*: *S* ≥ 11, *R* ≤ 10 mm; No. I category
Trimethoprim–sulphamethoxazole (1.25/23.75 µg)	25	31	20	20	28	27	**NI**; no applicable rabbit‐specific *P. multocida* DD CBP
Enrofloxacin (5 µg)	27	40	29	30	20	24	Rabbit CBP is unavailable. Reference only: cattle respiratory *P. multocida*: *S* ≥ 21, *I* 17–20, *R* ≤ 16 mm; swine: *S* ≥ 23, I 19–22, *R* ≤ 18 mm
Cefazolin (30 µg)	29	33	26	25	30	25	**NI**; no applicable rabbit‐specific *P. multocida* DD CBP
Ceftizoxime (30 µg)	38	44	35	35	40	35	**NI**; no applicable rabbit‐specific *P. multocida* DD CBP

*Note*: Values are inhibition‐zone diameters in millimetres. Rabbit‐specific clinical breakpoints for *P. multocida* are not available. Where CLSI VET01S provides clinical disk‐diffusion breakpoints for *P. multocida* respiratory infections in cattle or swine, these are shown for reference only and were not applied to classify the rabbit isolates as clinically susceptible or resistant. ^*^ *A value of 0 indicates no measurable inhibition zone beyond the antimicrobial disk*.

Abbreviations: DD, disk diffusion; CBP, clinical breakpoint; MIC, minimum inhibitory concentration; NI, not interpreted.

The observed inhibition‐zone ranges were 15–32 mm for penicillin G, 29–44 mm for ceftriaxone, 25–36 mm for tetracycline, 12–28 mm for erythromycin, 30–38 mm for florfenicol, 32–50 mm for lincomycin–spectinomycin, 22–36 mm for ampicillin, 10–23 mm for streptomycin, 10–29 mm for ceftiofur, 0–16 mm for tilmicosin, 20–31 mm for trimethoprim–sulphamethoxazole, 20–40 mm for enrofloxacin, 25–33 mm for cefazolin and 35–44 mm for ceftizoxime. Four isolates showed no measurable inhibition zone around the tilmicosin disk. CLSI disk‐diffusion breakpoints available for *P. multocida* respiratory infections in cattle or swine are provided in Table 6 only as non‐rabbit reference criteria.

## 4 Discussion

*P. multocida* remains one of the most important bacterial pathogens of rabbits, causing conditions ranging from asymptomatic colonization of the upper respiratory tract to severe respiratory infections, abscess formation, septicaemia and death. Because companion rabbits are increasingly maintained in close contact with humans, characterization of circulating *P. multocida* strains has become increasingly important from both veterinary and One Health perspectives (Harper et al. 2006; Gleeson and Petritz 2020; Wilson and Ho 2013; Hasani et al. 2024).

In the present study, *P. multocida* was confirmed in six of 65 sampled companion rabbits (9.23%). Most animals in the study population (62/65, 95.4%) had no apparent clinical abnormalities at the time of sampling, whereas three rabbits presented concurrent clinical findings. Notably, all three rabbits with recorded clinical abnormalities were *P. multocida*‐positive. Among the six PCR‐positive rabbits, three had no apparent clinical signs and can therefore appropriately be considered asymptomatic carriers. One positive rabbit had ocular and nasal discharge accompanied by respiratory signs, whereas two positive rabbits had dental malocclusion with dental abscesses. The latter three animals should not be described as asymptomatic carriers. Ocular and nasal discharge, respiratory signs and abscess formation may overlap with recognized clinical manifestations of rabbit pasteurellosis. However, because the present investigation was cross‐sectional and was not designed to establish the aetiology of individual clinical abnormalities, a causal relationship between *P. multocida* detection and these findings cannot be confirmed. Accordingly, these clinical abnormalities are reported descriptively rather than being directly attributed to, or considered unrelated to, *P. multocida*.

The observed frequency of *P. multocida* detection in the sampled population should also be interpreted in the context of the recruitment strategy. Rabbits were recruited from several accessible sources, including a veterinary teaching hospital, local pet shops and university‐associated animal populations, rather than through probability‐based sampling of the overall companion‐rabbit population of Ahvaz or southwest Iran. Consequently, the observed frequency of 9.23% should not be interpreted as a population‐representative estimate of regional prevalence.

Previous studies have reported widely varying frequencies of *P. multocida* detection in rabbits. The frequency observed in the present sampled population was lower than those reported in some studies from Brazil and China but remained within the broad range previously described in rabbits (Ferreira et al. 2016; Wang et al. 2019; Mahrous et al. 2022; Tayeb et al. 2004). Direct comparisons among studies should nevertheless be made cautiously because reported frequencies may be influenced by differences in clinical status, source population, housing and management conditions, sampling strategy, geographical setting and diagnostic methodology. The present findings therefore provide baseline information on the occurrence of *P. multocida* within the sampled rabbit population, whereas larger population‐based studies using probability sampling would be required to estimate regional prevalence reliably.

No statistically significant association was observed between *P. multocida* isolation and age, sex or breed. Nevertheless, five of the six positive rabbits were younger than 1 year of age. Given the small number of positive animals and the absence of a statistically significant association, this finding should be interpreted as a descriptive observation rather than evidence of an age‐related predisposition. Previous studies have suggested that colonization with *P. multocida* may become established early in life, and naturally infected rabbit colonies have shown acquisition of the organism during the first months of life (DiGiacomo et al. 1983; Percy and Barthold 2013; Gleeson and Petritz 2020; Yang et al. 2022). The predominance of younger animals among the positive rabbits in the present study is therefore compatible with these previous observations, although the current sample size was insufficient to determine whether age independently influences *P. multocida* detection.

Capsular typing demonstrated that five of the six isolates (83.3%) belonged to capsular type A, whereas one isolate remained NT by multiplex PCR. This finding agrees with previous investigations demonstrating that capsular type A is the predominant genotype among rabbit‐derived *P. multocida* isolates worldwide (Massacci et al. 2018; Ferreira et al. 2012; Ferreira et al. 2016; Mahrous et al. 2022; Tayeb et al. 2004; D'Amico et al. 2024). Likewise, Massacci et al. (2018) reported that most rabbit isolates belonged to capsular type A, reinforcing the strong association between this capsular genotype and the rabbit host. The predominance of capsular type A therefore appears to be highly conserved among rabbit‐associated strains despite geographical differences.

A particularly interesting finding of the present study was that all isolates belonged to LPS genotype L2. Previous studies have shown that rabbit isolates are most commonly associated with LPS genotype L3, whereas other LPS genotypes have been reported less frequently (Harper, John, et al. 2015; Harper, St Michael, et al. 2015; Massacci et al. 2018). Massacci et al. (2018) similarly reported a predominance of L3 among rabbit isolates, whereas Wang et al. (2019) identified different LPS profiles among respiratory isolates from rabbits in China. Therefore, the exclusive detection of L2 in the present study may represent regional variation among circulating *P. multocida* strains in southwest Iran rather than the globally predominant rabbit genotype. Nevertheless, because only six isolates were characterized, additional studies involving larger rabbit populations from different regions of Iran are needed to determine whether L2 is truly the predominant genotype in this geographical area.

Virulence profiling revealed that all isolates carried *sodA, exbD‐tonB, nanB* and *sodC*, suggesting that genes involved in oxidative stress resistance, iron acquisition and sialic acid metabolism are highly conserved among rabbit‐associated strains. Similar findings have been reported by Ewers et al. (2006), Ferreira et al. (2012) and Massacci et al. (2018), who identified these genes as part of the core virulence repertoire of *P. multocida* isolated from rabbits. In contrast, *ompH, hgbB, pfhA and nanH* were detected in 66.7% of isolates, whereas *ptfA, oma87, hgbA and toxA* were present in 50%, demonstrating considerable variability in accessory virulence determinants despite the relatively conserved capsular and LPS genotypes.

Among these virulence determinants, the detection of toxA deserves particular attention. The *toxA* gene encodes the dermonecrotoxin *Pasteurella multocida* toxin, which has classically been associated with porcine isolates causing progressive atrophic rhinitis (Lichtensteiger et al. 1996; Peng et al. 2019). Previous investigations involving rabbit‐derived *P. multocida* generally reported that *toxA* was absent or detected only rarely (Ferreira et al. 2012; Massacci et al. 2018; Mahrous et al. 2022). Likewise, Massacci et al. (2018) did not identify *toxA* as a common characteristic of rabbit isolates. Therefore, detection of *toxA* in 50% of the isolates in the present study represents an unusual finding and may indicate regional genomic diversity or the circulation of distinct rabbit‐associated lineages in southwest Iran. Because the present study relied on PCR detection only, future investigations employing multilocus sequence typing (MLST) and whole‐genome sequencing (WGS) are required to clarify the genetic background and epidemiological significance of these *toxA*‐positive isolates.

AST revealed considerable variation in inhibition‐zone diameters among the antimicrobial agents evaluated. However, interpretation of these findings requires caution. Host‐ and infection‐specific clinical breakpoints for *P. multocida* isolated from rabbits are currently unavailable, and clinical breakpoints established for other animal species cannot be assumed to predict therapeutic response in rabbits. Clinical breakpoint development incorporates not only the bacterial species and antimicrobial agent but also the host species, infection site, approved dosage regimen, route of administration and relevant pharmacokinetic/pharmacodynamic parameters (CLSI 2024a; Nielsen et al. 2021; Krüger‐Haker et al. 2026). Accordingly, the present antimicrobial susceptibility findings are primarily presented as quantitative in vitro inhibition‐zone measurements rather than as rabbit‐specific susceptible, intermediate or resistant categories. For selected antimicrobial agents, including ceftiofur, florfenicol, enrofloxacin and tilmicosin, CLSI provides disk‐diffusion clinical breakpoints for *P. multocida* associated with respiratory disease in cattle and/or swine; however, these criteria are provided in Table 6 only as non‐rabbit reference values and were not used to establish clinical susceptibility or resistance in the isolates examined here. For several other agents, an applicable disk‐diffusion clinical breakpoint is unavailable; notably, CLSI criteria for ampicillin, penicillin G and tetracycline in relevant bovine or porcine *P. multocida* respiratory infections are based on minimum inhibitory concentration (MIC) rather than disk‐diffusion zone diameters (Krüger‐Haker et al. 2026).

Direct comparison of the present inhibition‐zone data with previously published antimicrobial susceptibility studies of rabbit‐derived *P. multocida* is also difficult because of differences in study populations, antimicrobial panels, laboratory methods and interpretive criteria. Ferreira et al. (2012), using disk‐diffusion testing interpreted according to the CLSI standard applicable at the time, reported resistance among 46 rabbit‐derived *P. multocida* isolates most frequently to sulphonamides/trimethoprim–sulphamethoxazole (28.3%), followed by penicillin (10.9%), amoxicillin (6.5%) and erythromycin (4.3%), whereas all isolates were categorized as susceptible to ceftiofur, florfenicol, enrofloxacin and tetracycline. Similarly, Bourély et al. (2019), in a large French antimicrobial‐resistance surveillance study, reported generally low resistance proportions among rabbit‐derived *P. multocida* isolates. However, these studies used interpretive systems and epidemiological settings that differ from those of the present study. In particular, previously published categorical susceptibility or resistance percentages cannot be directly compared with the raw inhibition‐zone measurements reported here in the absence of validated rabbit‐specific clinical breakpoints. This limitation is consistent with the EFSA assessment of antimicrobial resistance in rabbit pathogens, which emphasized that comparisons among studies may be affected by differences in AST methods, interpretive criteria, study populations and the treatment of intermediate isolates in resistance calculations (Nielsen et al. 2021).

Nevertheless, the present inhibition‐zone measurements demonstrate clear differences in in vitro activity among the antimicrobial agents tested. Relatively large inhibition zones were observed with several agents, including florfenicol, whereas tilmicosin produced small or absent inhibition zones in several isolates. These observations should not, however, be interpreted as evidence of clinical therapeutic efficacy or treatment failure in rabbits. Antimicrobial susceptibility measured in vitro represents only one component of therapeutic decision‐making, particularly in rabbits, in which antimicrobial selection must also consider drug safety, gastrointestinal effects, route of administration, dosage, pharmacokinetics, infection site and the clinical condition of the individual animal. Therefore, the present results provide descriptive baseline information on the in vitro antimicrobial response of the isolates but should not be used alone to recommend specific antimicrobial agents for the treatment of rabbit pasteurellosis. Further studies incorporating larger isolate collections, MIC determination, pharmacokinetic/pharmacodynamic data and rabbit‐specific clinical outcome data are needed to establish clinically meaningful interpretive criteria for *P. multocida* in this host.

The detection of *P. multocida* in three clinically asymptomatic rabbits demonstrates that asymptomatic carriage occurred within the sampled population. Such clinically silent carriage may contribute to bacterial persistence and transmission within rabbit populations. However, because the remaining three PCR‐positive rabbits had concurrent clinical abnormalities, the entire group of positive animals should not be regarded as asymptomatic carriers. Moreover, because *P. multocida* is an established zoonotic pathogen, close contact between rabbits and their owners may facilitate transmission, particularly in immunocompromised individuals (Wilson and Ho 2013; Oehler et al. 2009; Hasani et al. 2024). Human infections associated with companion animals have been reported following bites, scratches or direct exposure to respiratory secretions, highlighting the importance of owner education, appropriate hygiene and routine veterinary monitoring.

The present study provides one of the first comprehensive molecular characterizations of *P. multocida* isolated from companion rabbits in Iran by combining species confirmation, capsular typing, LPS genotyping, virulence profiling and AST. Nevertheless, several limitations should be acknowledged. The number of positive isolates was relatively small, samples originated from a single geographical region and genomic analyses such as MLST or WGS were not performed. The small number of *P. multocida*–positive isolates also precluded meaningful statistical evaluation of associations between individual virulence genes and host characteristics. In addition, the rabbits were recruited from a limited number of accessible sources using a non‐population‐based sampling strategy; therefore, the observed detection frequency should not be generalized to the prevalence of *P. multocida* in the overall companion‐rabbit population of Ahvaz or southwest Iran. Future multicentre studies incorporating genomic epidemiology and larger sample sizes will provide a more comprehensive understanding of the evolution, dissemination and pathogenic potential of rabbit‐associated *P. multocida* strains in Iran and neighbouring regions.

## 5 Conclusion

This study provides baseline data on the molecular characteristics of *P. multocida* isolated from companion rabbits in southwest Iran. *P. multocida* was detected in rabbits with differing clinical presentations, including three clinically asymptomatic animals as well as three animals with concurrent clinical abnormalities. These findings emphasize the importance of distinguishing asymptomatic carriage from bacterial detection in animals with potentially compatible clinical signs. Most isolates belonged to capsular type A, and notably, all were classified as LPS genotype L2, a profile that differs from the predominant LPS genotypes previously reported in rabbits and may reflect regional variation among circulating strains. Virulence profiling demonstrated the widespread presence of genes associated with oxidative stress resistance, iron acquisition and host colonization, whereas the detection of the *toxA* gene in half of the isolates suggests potential genetic diversity that warrants further investigation using MLST and WGS. Disk‐diffusion testing demonstrated heterogeneous in vitro inhibition‐zone patterns among the antimicrobial agents examined. Because rabbit‐specific clinical breakpoints for *P. multocida* are unavailable, these findings should not be interpreted as direct evidence of clinical susceptibility, resistance or therapeutic efficacy in rabbits. Reporting the individual inhibition‐zone diameters provides a transparent baseline for future comparison and re‐evaluation as rabbit‐specific interpretive criteria become available. Collectively, these findings expand current knowledge of the molecular characteristics of *P. multocida* circulating in clinically healthy companion rabbits and highlight the need for continued molecular surveillance within a One Health framework to improve disease monitoring, guide antimicrobial use and better understand the epidemiology of rabbit‐associated *P. multocida*.

## Author Contributions

Darioush Gharibi conceived and supervised the study, designed the methodology, interpreted the data and drafted and critically revised the manuscript. Mahdi Pourmahdi Borujeni contributed to data interpretation and critically reviewed the manuscript. Bahman Mosallanejad contributed to veterinary examination of the rabbits, study coordination and manuscript revision. Fatemeh Sadat Asadi Khoonsari collected the samples, performed bacteriological and molecular laboratory analyses, conducted the antimicrobial susceptibility testing and analysed the data. All authors read and approved the final version of the manuscript.

## Funding

This research was funded by the research grant number (SCU.VP1403.261) from Shahid Chamran University of Ahvaz.

## Ethics Statement

This study involved non‐invasive collection of nasal and oropharyngeal swab samples from companion rabbits. The study protocol was reviewed and approved by the Ethics Committee of Shahid Chamran University of Ahvaz (Ethics approval code: **EE/1401.2.24.227342/scu.ac.ir**). All sampling procedures were performed by a licensed veterinarian in accordance with institutional guidelines and internationally accepted principles for the ethical use of animals in research, including the International Council for Laboratory Animal Science (ICLAS) Ethical Guidelines. Informed consent was obtained from the animal owners prior to sample collection.

## Conflicts of Interest

The authors declare no conflicts of interest.

## Supporting information




**Supporting File**: vms371323‐sup‐0001‐SuppMat.pdf

## Data Availability

The data that support the findings of this study are available from the corresponding author upon reasonable request.
