# Supplementary material for: Molecular Characterization, Virulence Gene Profiling and Antimicrobial Susceptibility of Pasteurella multocida Isolated From Companion Rabbits in Southwestern Iran
Source: Vet Med Sci. 2026 Nov 12;12(6):e71323. doi: 10.1002/vms3.71323 (PMC13759009; doi:10.1002/vms3.71323)
Supplement: Supplementary file 1 [file VMS3-12-e71323-s001.pdf]

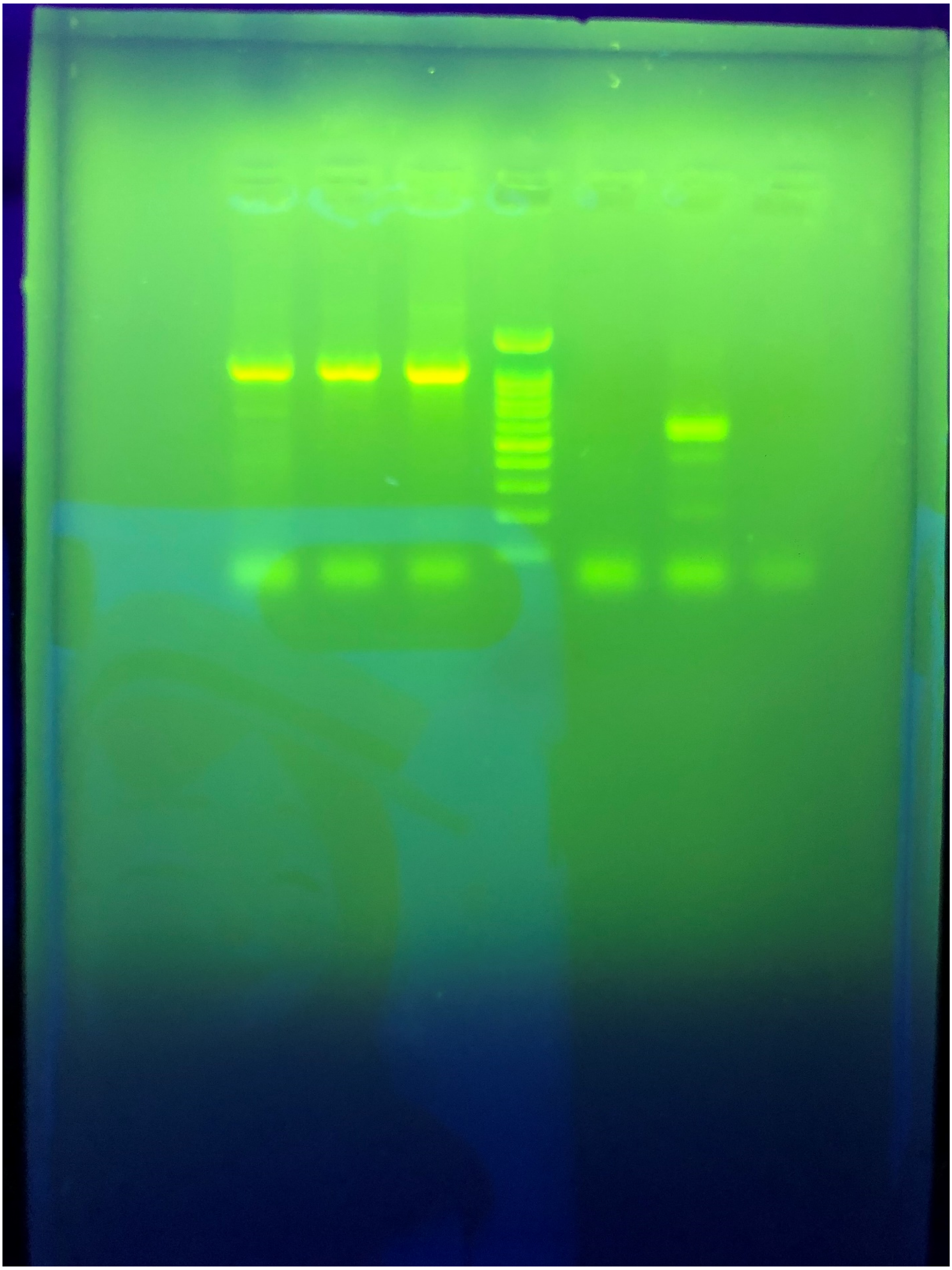

Capsular typing

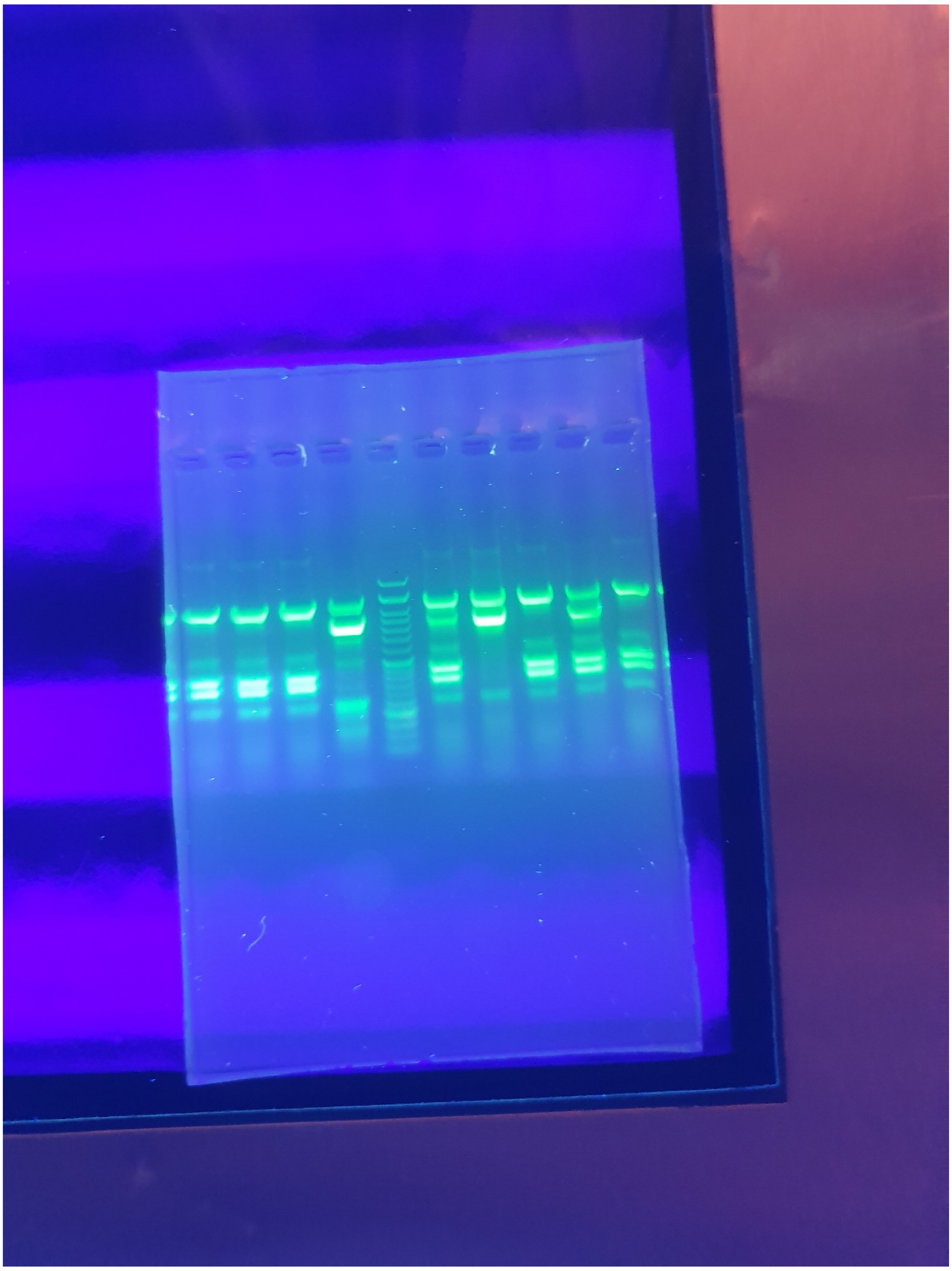

exbD-tonB, toxA, ompH, and nanH

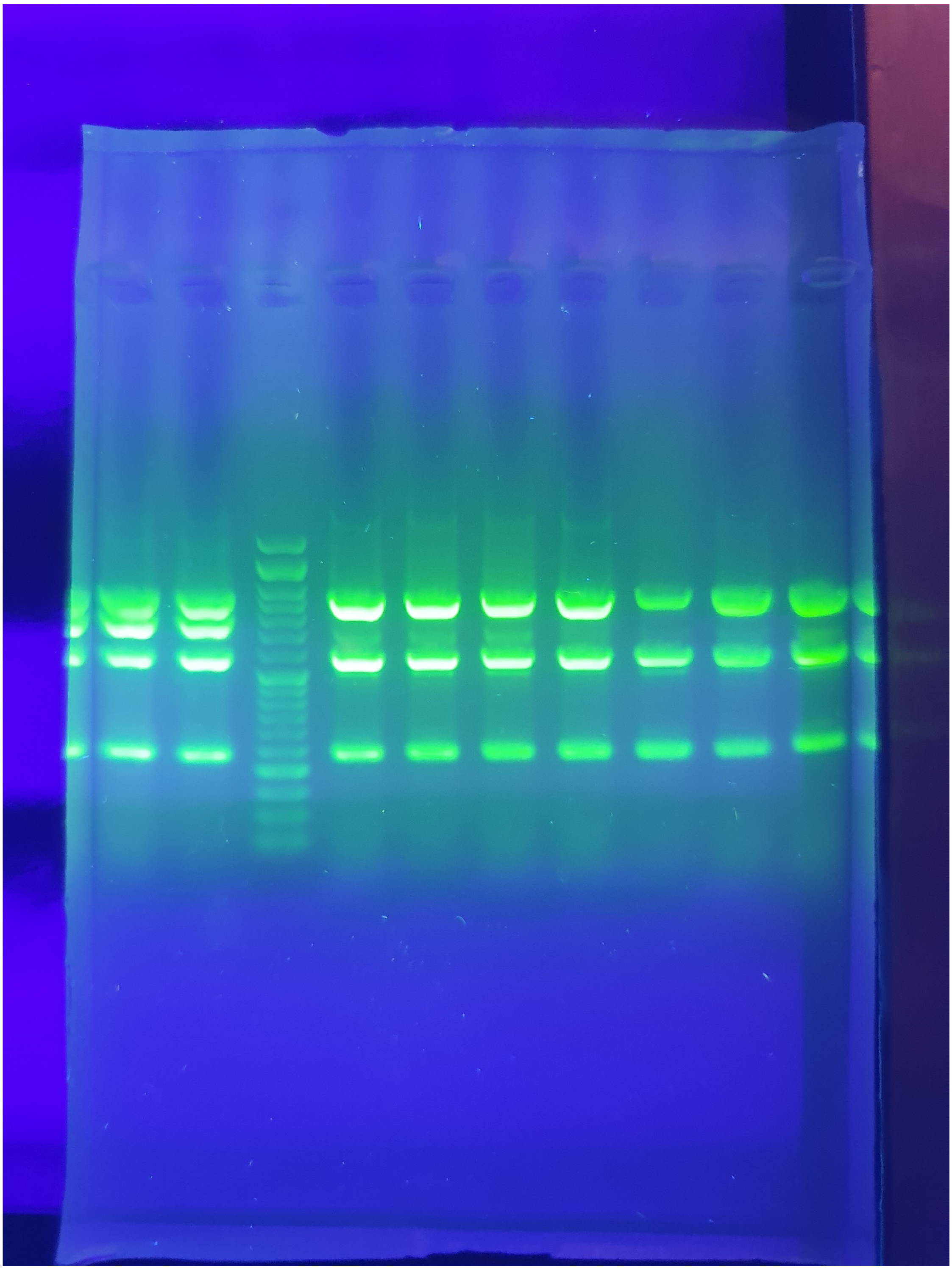

oma87, hgbB, nanB, and sodC

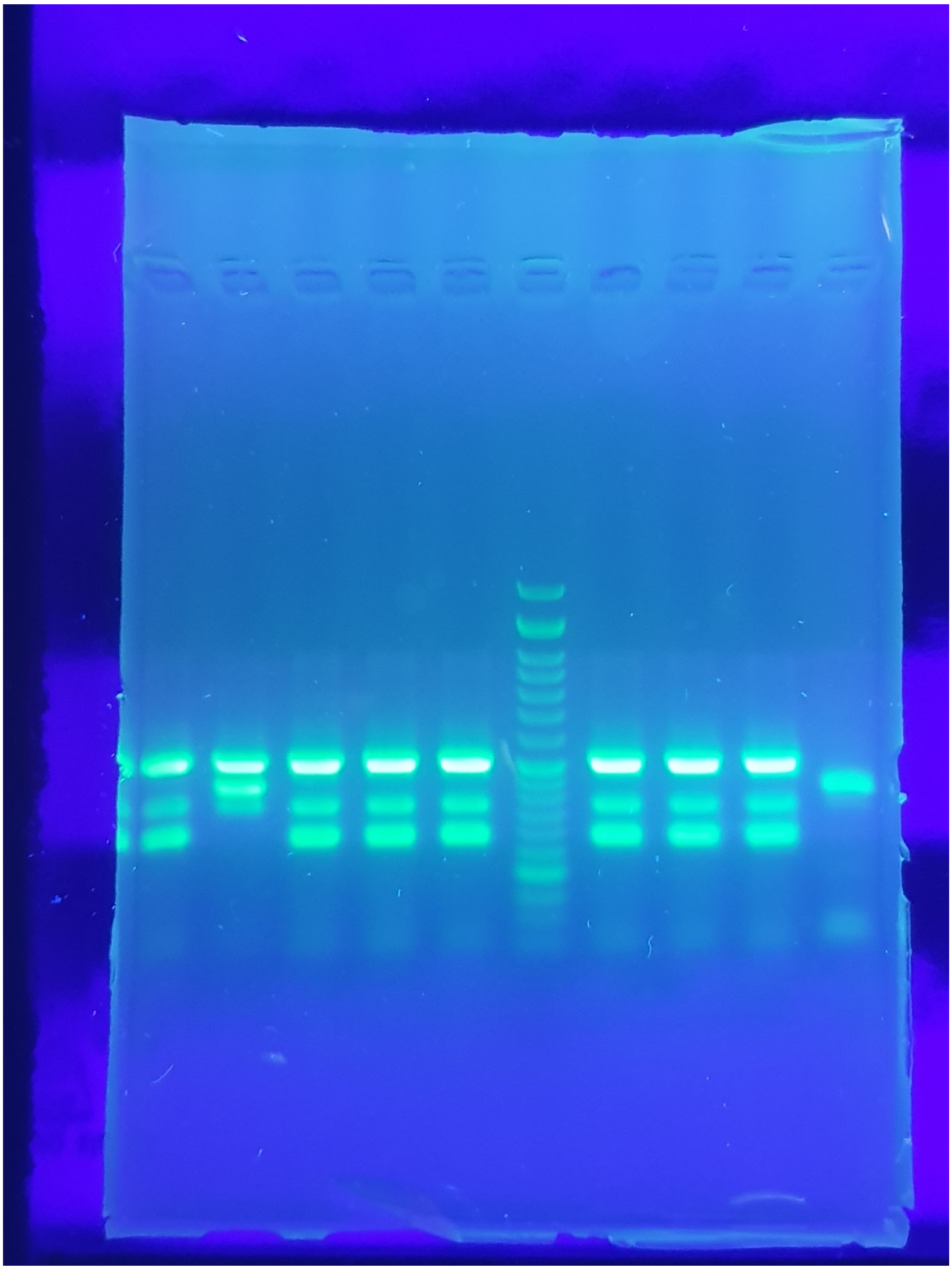

PtfA, SodA, PfhA, HgbA

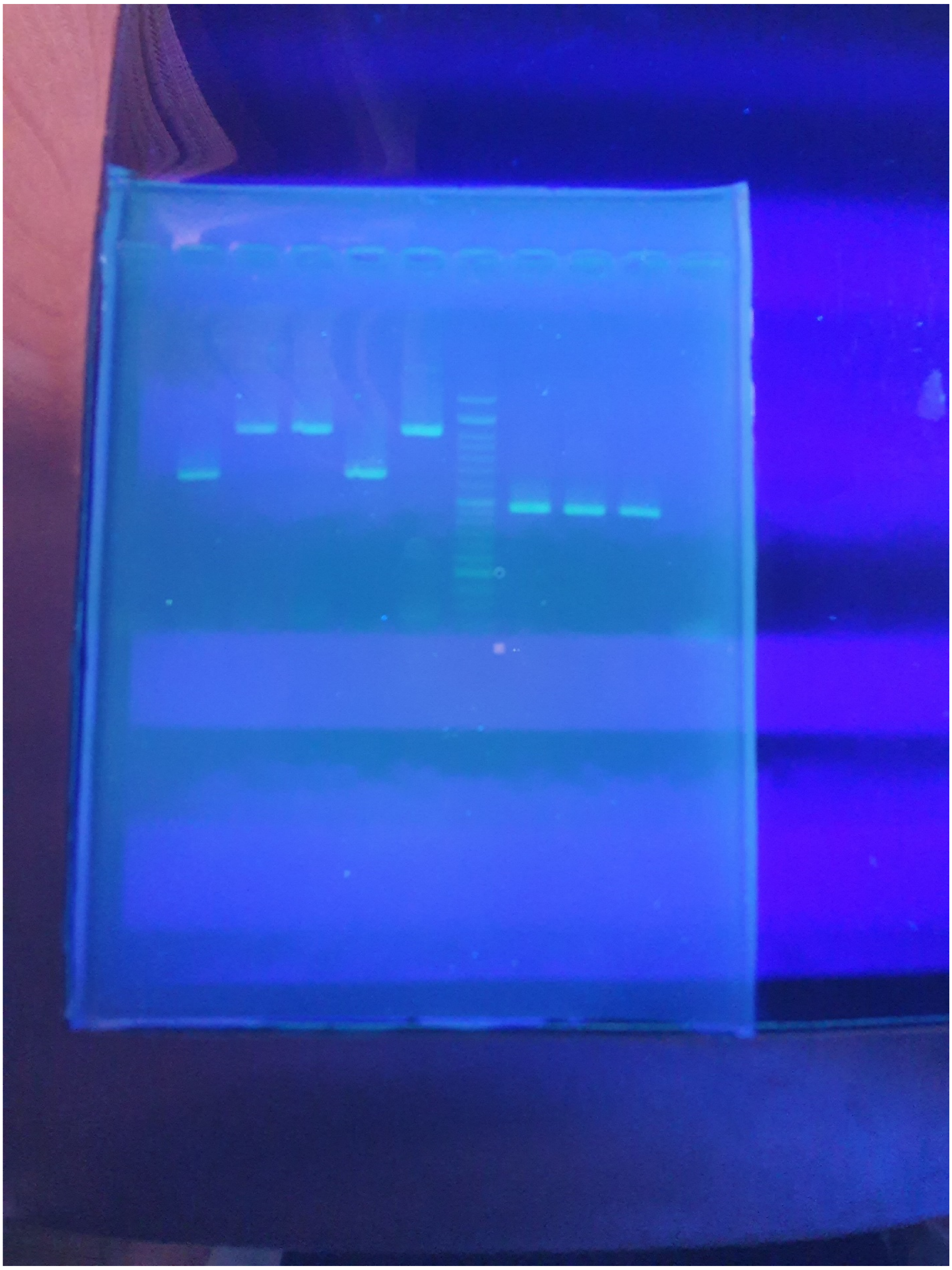

KMT1

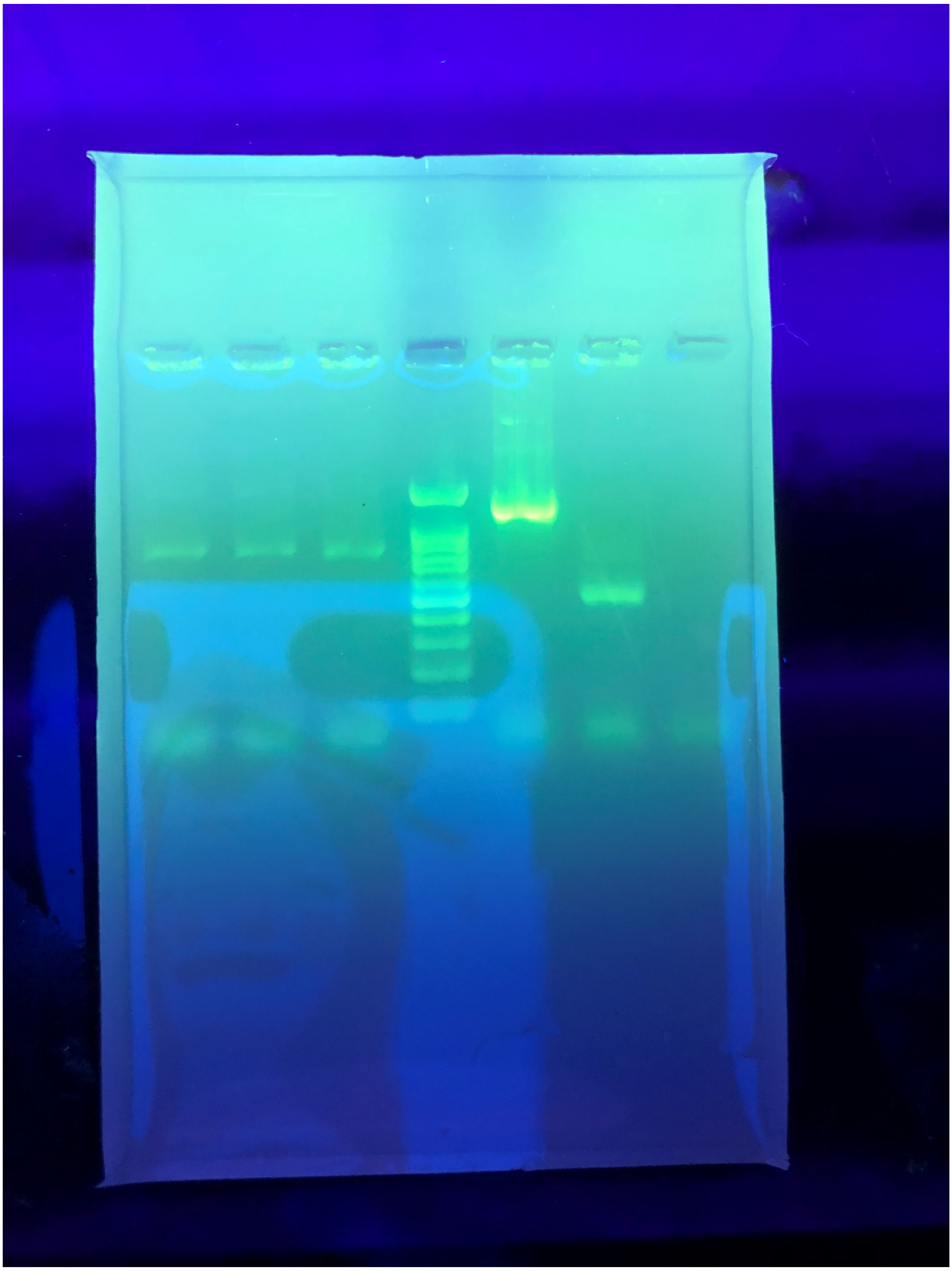

LPS type
